# Effectiveness and tolerability of a squalane and dimethicone-based treatment for head lice

**DOI:** 10.1007/s00436-021-07113-y

**Published:** 2021-04-02

**Authors:** Leticia Martínez de Murguía Fernández, Gemma Puig Algora, Marta Bajona Roig, Gabriela Bacchini

**Affiliations:** 1 TECNALIA, Basque Research and Technology Alliance (BRTA), Área Anardi, 5, E-20730 Azpeitia, Gipuzkoa Spain; 2 Ferrer, Cosmetic Development, C/Joan XXIII, 10, E-08950 Esplugues de Llobregat, Spain; 3 Ferrer, Corporate Medical Affairs Department, Av. Diagonal 549, E-08029 Barcelona, Spain

**Keywords:** Head lice, Pediculicides, Physically acting, Ovicidal, In vitro efficacy, Children

## Abstract

Head lice (*Pediculus humanus capitis*) are worldwide obligate human ectoparasites, with high implications in pediatrics. In Europe, first-line topical neurotoxic insecticidal therapeutic strategies are being replaced by topical physically acting agents as the first-choice treatment. Safety of the active ingredients and high efficacy in a one-time single-dose treatment with a brief treatment application time are key issues for consumer use and effective compliance. The aim of this study was to assess the in vitro efficacy of a newly developed squalane and dimethicone-based pediculicidal formula, against motile head lice and eggs after 2 and 5 min immersion in the product, as well as its skin tolerance and acceptability under dermatological and pediatric expert control in children with atopic skin. The results indicate that at both time points, 100% mortality rate of head lice crawling stages and late-stage eggs was achieved. The formula was well tolerated and suitable for children with atopic skin from 12 months of age. Showing high in vitro efficacy and good skin acceptability, this solution is presented as a new safe alternative therapy for treatment of head lice infestations.

**Supplementary Information:**

The online version contains supplementary material available at 10.1007/s00436-021-07113-y.

## Introduction

Head louse pediculosis due to *Pediculus humanus capitis* De Geer, 1778 is an endemic human ectoparasitic disease worldwide (Falagas et al. 2008). With variable infestation rates among regions and countries, it is considered a public health and social problem (Coates et al. 2020; Falagas et al. 2008; Feldmeier 2012) with economic implications (Hansen and O’Haver 2004; West 2004). Although all ages and diverse socioeconomic backgrounds are affected, it is particularly frequent among school children aged 4 to 12 years and other vulnerable groups such as the homeless, orphans, geriatrics, and refugees (Dietrich et al. 2018; Meister and Ochsendorf 2016; Tytuła et al. 2019). Pruritus, scalp irritation with potential secondary bacterial infections (Chosidow 2000) and social and psychological distress (Gordon 2007) are the main clinical manifestations. Unlike body lice, and despite identification on head lice of human pathogens including trench and relapsing fever bacteria, they are not known to transmit infectious diseases (Amanzougaghene et al. 2020).

Different head louse treatments based on diverse mechanisms of action are currently available (Sangaré et al. 2016). However, traditional topical neurotoxic insecticides are under review; some active ingredients have been banned based on safety concerns, while others have lost efficacy due to increasing resistance of head louse populations in different countries (Clark et al. 2015; Durand et al. 2012; Gellatly et al. 2016). As a result, new active ingredients and different treatment approaches are being developed around the world (Burgess 2016). In Europe, although safe formulations of insecticides (pyrethrins and pyrethroids) remain as first-line therapy, physically acting treatments are now gaining ground as the first-choice treatment (Burgess 2018, Burgess 2016; Feldmeier 2014; Meister and Ochsendorf 2016). Among these, several different products based on silicones, fixed and mineral oils, lipid esters, surfactants, or oil-surfactant copolymers have been commercialized (Burgess 2018; Burgess 2016). Nevertheless, not all active ingredients and formulations show ovicidal activity, and a second or third treatment at 7- to 10-day intervals is often required to prevent emerging nymphs from establishing a new infestation (Barker et al. 2012; Lebwohl et al. 2007). For users, low compliance with this treatment strategy leads to treatment failure (Meister and Ochsendorf 2016). Thus, the development of new alternative products that target eggs and motile stages simultaneously, to treat head louse infestations in a single-dose treatment, is strongly encouraged (Barker et al. 2012).

In this context, a new innovative physically acting product was developed to be used in one single dose. The aim of the studies presented here was to evaluate the in vitro adulticidal and ovicidal efficacy of a new squalene and dimethicone-based product in different short immersion exposures and to assess its skin tolerance and acceptability under dermatological and pediatric expert control in children with atopic skin.

## Material and methods

### Head lice and ethics

Head lice were collected from the heads of volunteers by specialized personnel. Volunteers had not been treated with anti-louse products for 4 weeks before specimen collection. Fine-tooth lice combs were used for nymphs and adult lice collection, and only those eggs grey in color and less than 1 cm from the scalp were obtained by cutting the hair shaft at the base with round-pointed scissors. Volunteers gave written informed consent following the protocol approved by the Clinical Research Ethics Committee of the Hospital of Donostia (Gipuzkoa, Spain).

### Products

Studies were conducted with the ready-to-use product “OTC ANTIPIOJOS FORMULA TOTAL,” provided by Ferrer International S.A. (Spain). It is formulated with squalane, dimethicone, and lauryl alcohol. The formula contains a mix of low viscosity, volatile dimethicone, and medium viscosity dimethicone together with a 4% of high viscosity dimethicone. Squalane used in this formula is found in high concentration, and it comes entirely from bio-based feedstock: plant sugars, such as sugarcane. References “Ref. 72.1” and “Ref. 72.4” are two replicates of this final formulation, used to conduct the in vitro efficacy studies. The clinical study to assess skin tolerance was conducted with “Ref. 72.4.”

### In vitro efficacy testing

Both motile or crawling head lice and eggs were used. Product samples were conditioned at the same environmental conditions as the treatment application: 25 ± 2 °C and 60 ± 10% relative humidity (RH). Treatments were carried out in 10 ml of product poured into 5-cm diameter Petri dishes following an in vitro immersion methodology (Mougabure Cueto et al. 2000), by submerging each group of specimens in the product. Specimens were then transferred to a permeable well, rinsed with tap water for 1 min., and settled in 5-cm Petri dishes, previously labeled and lined with moistened filter paper (Whatman no. 1). Tap water was used in the negative controls, which were similarly conditioned. All experiments were done in triplicate.

Groups of 10 specimens of motile stages (3rd nymphal instar and adults of both sexes) were treated between 3 and 5 h after collection by immersion in the product for 2 min and incubated in the dark at 25 ± 2 °C and 60 ± 10% RH until time point observations. Vital signs were evaluated at 5, 15, 30, 60, and 180 min and 18 h post-product application under a stereoscopic microscope (Olympus SZ61TR with zoom up to 4.5 × 20 magnifications) and following strictly defined criteria for vitality (Table 1). Mortality is the result of the combination of knocked down (vital signs reduced [VSR]) and dead (D) lice.

**Table 1:** Defined vitality criteria in motile lice and ova for product efficacy evaluation

Motile head lice vital signs’ classification	
(i) Alive	Lice can walk, grab a hair, and move forward
(ii) Vital signs apparent (VSA)	Lice movements are not coordinated, and they cannot walk or grab a hair and move forward
(iii) Vital signs reduced (VSR)	Lice show small movements in the antennae, legs, or digestive tract
(iv) Dead (D)	Lice show complete cessation of movement, including the digestive tract
Ova vital signs’ classification	
(i) Complete hatching (CH)	Eggs with the operculum opened and empty
(ii) Incomplete hatching (IH)	Eggs with the operculum opened, but the nymph remains entirely or partially inside
(iii) No hatching of late stages (LS)	Eggs with the operculum closed and dead embryo inside

Classification of motile head lice vital signs according to Oliveira et al. (2007)

Hair shafts with eggs were inspected under the stereoscopic microscope and classified into early (no differentiation) and late (red or black eye spot and appendages and/or embryonic movements) stages, according to Sonnberg et al. (2010). Only late-stage eggs were used in this study. Groups of 10 specimens on hair shafts (3 cm) were treated by immersion at two different time exposures, 2 and 5 min. They were then incubated in the dark at 29 ± 1 °C and ˃70 % RH until observation, at 7 days post-treatment, when all eggs in the controls had hatched. Defined criteria for viability are shown in Table 1. Mortality is the result of the combination of incomplete hatching (IH) and no hatching of late stages (LS).

### Statistical analysis

The results from the replicates were pooled, and data was presented as the arithmetic mean percent adulticidal and ovicidal activity with their corresponding standard deviations (± SD). The efficacy outcome was the mortality rate within each group of specimens considering both the number of alive and affected lice (VSR + D) or completely hatched (CH) and affected eggs (IH + LS) according to the equation (Number of affected lice or eggs/Total number of lice or eggs) × 100. For final values, correction considering mortality in the controls was applied with the formula ([mortality % treatment – % mortality control/100- % mortality control] × 100). Statistical comparison of mortality relative frequencies in the treatments and controls were conducted using the Chi-squared test with the software package PAST (Hammer n.d., Version 4.02; Hammer et al. 2001).

### Clinical assessment of skin tolerance and acceptability under normal use conditions

A clinical, prospective study was conducted to assess the absence of adverse reactions of discomfort and cumulative irritations (functional and physical signs) related to the application of the product in normal use conditions under dermatological and pediatric monitoring, as well as its acceptability, following the corresponding protocol and according to Good Clinical Practice guidelines in San Fermín Clinic (Pamplona, Spain). One pediatrician and one dermatologist assessed the clinical tolerance of the product by visual evaluation.

Children with atopic skin aged between 1 and 3 years, no flare-up in the last 4 weeks, were included after agreement and informed written consent by parents or guardians. The sample size was 20 evaluable children; this number being considered sufficient to assess the safety of the product by the investigators.

The parents/guardians of the children made three visits to the clinic in a 14-day period: day 0, day 7, and day 14. On days 0 and 7, the pediculicide lotion was applied to the dry hair of the children under normal conditions of use, shown in Table 2. At all visits, the researchers evaluated the area of application of the product to assess possible adverse reactions or irritation produced in the scalp, hair, and skin and especially in the eyes. Parents/guardians were also contacted by telephone within 3–4 days of application of the study product to confirm the absence of any adverse reactions. At the last visit, parents/guardians were asked to complete a 4-point subjective product evaluation questionnaire to assess its organoleptic properties, where 0 is strongly disagree, 1 is disagree, 2 is agree, and 3 is strongly agree. The results were expressed as % of parents/guardians satisfied with the product (score of 2 or 3).

**Table 2:** Instructions for application of the product “OTC ANTIPIOJOS FORMULA TOTAL” under normal conditions of use

Step	Instruction
1	Unlock the spray safety; hold the spray 10 cm from the hair, and spray sufficient product onto dry hair
2	Gently massage so that the hair and scalp are well saturated
3	Leave on the hair for 2 min
4	Comb the hair with the nit comb from the root to the tips
5	To remove the product, apply the shampoo directly on the hair after the treatment without first wetting or rinsing it (since the product repels water)
6	Shampoo the hair thoroughly and rinse afterwards with plenty of water. If necessary, repeat the wash to ensure that the entire product has been removed

## Results

### Adulticidal efficacy

Two-minute immersion treatment of crawling stages showed that at 5 min post-treatment, all lice were classified as non-viable, with only three lice showing few minor vital signs (gut movements) and the rest described as dead at this observation point (Table 3). Ten minutes later, all individuals were dead. Immobilization was observed in all individuals immediately after application, and gut rupture was observed at different time points. Results were similar in all three replicates and were pooled, giving a final mean mortality of 100 ± 0% at all time point observations, as compared with control lice that were all alive 3 h after treatment with water. The mean percentage mortality observed in the negative controls at 18 h (47%) was due to dehydration of the lice when they are outside the host. Mortality with the product was statistically significant from the water controls, at both endpoints, 3 h (Chi^2^ = 60, d.f. 1, *P* < 0.001) and 18 h (Chi^2^ = 21.818, d.f. 1, *P* < 0.001).

**Table 3:** Vital signs recorded for motile head lice

Treatment	Replicates	Time treatment application: 2 min					
		Time point observations after treatment application					
		5 min	15 min	30 min	60 min	180 min	18 h
Control	3 (*n* = 30)	0	0	0	0	0	14^b^
	Mortality (%) Mean ± SD	00 ± 0	00 ± 0	00 ± 0	00 ± 0	00 ± 0	47 ± 6
OTC ANTIPIOJOS FORMULA TOTAL “Ref. 72.1”	3 (n = 30)	3^a^ 27^b^	30^b^	30^b^	30^b^	30^b^	30^b^
	Mortality (%) Mean ± SD	100 ± 0	100 ± 0	100 ± 0	100 ± 0	100 ± 0	100 ± 0
Corrected mortality (%) Mean ± SD		100 ± 0	100 ± 0	100 ± 0	100 ± 0	100 ± 0	100 ± 0

^a^VSR (vital signs reduced); ^b^D (dead)

Raw data and mean percentage mortality (%) and standard deviations (SD) for each observation time point and pooled replicates after the 2-min immersion treatment with “OTC ANTIPIOJOS FORMULA TOTAL”

### Ovicidal efficacy

Both the 2- and 5-min immersion treatments with late-stage eggs showed similar results in all replicates and were pooled, being 100% the mean mortality value as no louse hatched after 7 days and hatching in the negative controls gave 83.3% survival, respectively (Table 4). Corrected mortality values confirm a mean of 100% efficacy in both treatment regimes. When comparing between the product and control, differences were statistically significant in the two-time experiments (Chi^2^ = 42.857, d.f. 1, *P* < 0.001). All eggs died within the stage they were treated, red or black eyespot, with no incomplete hatching or dead emerging nymph observed in any of the treated replicates. Observations made immediately after the 2-min treatment showed that periodic pumping movements of the pharynx and gut movements had ceased in several eggs but continued to be observed in other specimens. As we did not record this data, we do not know the exact moment when the embryos died.

**Table 4:** Vital signs recorded for head lice late-stage eggs

Treatment	Replicates	Reference and time treatment application					
		Ref. 72.1: 5 min			Ref. 72.4: 2 min		
		LS^c^	IH^d^	CH^e^	LS^c^	IH^d^	CH^e^
Control	3 (*n* = 30)	5	0	25	5	0	25
	Mean ± SD (%)	16.67 ± 5.77	00 ± 0.00	83.33 ± 5.77	16.67 ± 5.77	00 ± 0.00	83.33 ± 5.77
OTC ANTIPIOJOS FORMULA TOTAL	3 (*n* = 30)	30	0	0	30	0	0
	Mean ± SD (%)	100 ± 0.00	00 ± 0.00	00 ± 0.00	100 ± 0.00	00 ± 0.00	00 ± 0.00
Corrected mortality Mean ± SD (%)		100 ± 0.00	00 ± 0.00	-	100 ± 0.00	00 ± 0.00	-

^c^*LS* no hatching of late stage, ^d^*IH* incomplete hatching, ^e^*CH* complete hatching

Raw data and mean percentage mortality (%) in late stages (LS) and incomplete hatching (IH) categories, along with survival data (CH), with their standard deviations (SD) for pooled replicates and treatment exposure time (5 and 2 min) at 7 days post-product application with “OTC ANTIPIOJOS FORMULA TOTAL.” Survival in the controls corresponds to the mean natural egg hatching rate, reported to be 76% by Takano-Lee et al. (2003)

### Clinical assessment of skin tolerance and acceptability under normal use conditions

A total of 10 boys and 10 girls were included in the study, from 12 to 34 months old. The product did not produce any undesirable skin reactions in the study participants after 14 days of use, according to the study researchers (dermatologist and pediatrician). During the 14-day study period, none of the parents or guardians reported any undesirable reactions. Overall, 100% of parents or guardians said that they were satisfied with the properties of the product, including odor, texture, quick and easy application, absence of skin and eye irritation, or redness.

## Discussion

Physically acting pediculicides are a safe and effective alternative to traditional insecticides for treating head louse infestations (Burgess 2018; Burgess 2016; Clark et al. 2013; Feldmeier 2014; Flores-Genuino et al. 2020). A one-time single-dose treatment that simultaneously targets motile head lice and eggs on the scalp is easier to use and aims to increase cure rates (Abdel-Ghaffar et al. 2012; Barker et al. 2012; Burgess and Burgess 2011; Heukelbach et al. 2011; Semmler et al. 2017). Furthermore, efficacy with a short application time (5 min or less) is considered to fulfill the requirements of a first-choice pediculicide, which, in addition, should also avoid louse resistance development and be safe for both host and environment (Abdel-Ghaffar et al. 2010; Gordon 2007).

The specific composition of “OTC ANTIPIOJOS FORMULA TOTAL” is included in an International Application published under the Patent Cooperation Treaty (PCT), International Publication Number WO 2019/008116 (Bacchini and Puig Algora 2018). Squalane, a saturated aliphatic hydrocarbon, is obtained by the hydrogenation of squalene, which is contained in shark liver oil, rice, olives, or soybean (Popa et al. 2014). Squalane used in this formula is a product of fermentation and comes entirely from bio-based feedstock. It is certified as 100% bio-based (United States Department of Agriculture n.d.) and conforms to ECOCERT’s natural cosmetic standard (Ecocert Group n.d.). It adheres to the International Standard ISO 16128-1 (International Organization for Standardization n.d.-a) and has a natural origin index of 1.00 per ISO 16128-2 (International Organization for Standardization n.d.-b). Squalane has low toxicity and is frequently used in cosmetics as an emollient and moisturizer (Kelly 1999; Popa et al. 2014). Indeed, squalane emulsions have been proposed as viable alternative formulations in efficient vaccine delivery systems (Kantipakala et al. 2019; Kelly 1999). Dimethicone, a clear and odorless polydimethylsiloxane (PDMS) fluid with different molecular weights, is widely used in cosmetics, with a good safety profile (Becker et al. 2014), and in pharmaceutical products including head louse treatments (Colas et al. 2005). They are applied in the same way as other lotions for head louse infestation, by coating the scalp and full length of the hair (Burgess et al. 2005). They seem less irritant than existing pediculicidal treatments and have a physical action on lice that should not be affected by resistance to neurotoxic insecticides (Burgess 2018). Lauryl alcohol (1-dodecanol), a fatty alcohol produced from palm kernel oil or coconut oil and widely used as a liquid emulsifier and emollient for cosmetics formulations, is a desirable component in pediculicidal hair lotions for its effects on both motile lice (Mougabure Cueto et al. 2002) and eggs (Heukelbach et al. 2007). Previous studies on the mechanism of action with an initial formulation containing squalane, cyclopentasiloxane—a volatile silicone—and ethylhexyl stearate were performed (Bacchini and Puig Algora 2018; Bajona Roig et al. 2019a). They indicated that the squalane-based formula, without dimethicone, was able to penetrate through the cuticle of the lice exoskeleton and into the respiratory tract of motile stages (staining of spiracles, trachea, and tracheoles) and aeropyles of the operculum of late eggs (inner membranes surrounding the embryo were stained). Moreover, after 60–90 s of lice immersion in the formulation, any apparent movement stops besides peristaltic movements of the intestine, without subsequent signs of recovery. These findings, along with gut rupture in motile stages, are similar to those described for a physical mode of action of silicone-based products: disruption of the integrity of the lipid cuticle of the louse exoskeleton leading to dehydration and death (Barnett et al. 2012) and occlusion of the respiratory system causing death by osmotic stress due to inhibition of water excretion (Burgess 2009). Anoxia or suffocation, as the mode of action, is controversial (Burgess 2018). With respect to eggs, it has been suggested that effective compositions could act by blocking the micropyles of the inner membranes, interfering in the respiratory processes by limiting the oxygen supply or favoring carbon dioxide accumulation, leading in both cases to death of the embryo (Heukelbach et al. 2011; Mehlhorn et al. 2011).

The direct immersion methodology applied for the efficacy studies is considered a sensitive method to detect small differences in mortality between formulations (Gallardo et al. 2012). This claim is in line with the results obtained with different initial squalane-based formulations—without dimethicone—previously tested (Bacchini and Puig Algora 2018; Bajona Roig et al. 2018). Efficacy against crawling stages was consistently as high as 100% at different immersion times. However, the mean ovicidal mortality ranged from 27 to 93%, depending on the immersion time (2 to 12 min, respectively).

The final formulation used in this in vitro efficacy study has shown 100% adulticidal and ovicidal effectiveness. It contains a mix of low, medium, and high viscosity dimethicones, the latter in 4% concentration that has been shown to be effective in physical acting head louse treatments (Burgess et al. 2005). This carefully designed combination of dimethicones of different molecular weights together with high concentration of squalane and a small amount of lauryl alcohol improves its mortality potency and confers good cosmeticity. Its good efficacy is similar to the data reported in other studies of different silicone-based commercial pediculicides (Gallardo et al. 2012; Heukelbach et al. 2019). Furthermore, the formulation shows full efficacy in only 2 min, following the global tendency to reduce the application time in order to increase treatment compliance. Other in vitro studies of physical acting agents confirm that motile head lice and eggs can be killed within a few minutes, although not all formulations achieve 100% ovicidal activity (Abdel-Ghaffar et al. 2012; Abdel-Ghaffar et al. 2010; Gallardo et al. 2012; Heukelbach et al. 2019; Mehlhorn et al. 2011; Oliveira et al. 2007; Strycharz et al. 2012).

Good skin compatibility, even for use in young children with atopic skin, was obtained with the final formulation. These results are in line with previously reported data with the initial squalene-based formulation (Bacchini and Puig Algora 2018; Bajona Roig et al. 2019b). It offers the highly desirable combination of being effective, spreading evenly, drying quickly, and being hair and skin compatible by not having a greasy or oily texture. Therefore, it eliminates the disadvantages of previously available compositions, namely, being difficult and unpleasant to apply, emitting a nasty odor, having limited effectiveness, and having substantial mammalian toxicity or tolerability issues.

Thus, according to the studies, this new squalene and dimethicone-based pediculicide complies with the main characteristics to become an effective alternative therapy in one single and brief application dose for children from 12 months of age, with good skin compatibility and acceptability.

## Supplementary Information

ESM 1 (PDF 432 kb)

ESM 2 (PDF 915 kb)
