# Supplementary material for: Effectiveness and tolerability of a squalane and dimethicone-based treatment for head lice
Source: Parasitol Res. 2021 Apr 2;120(5):1883–90. doi: 10.1007/s00436-021-07113-y (PMC8084834; doi:10.1007/s00436-021-07113-y)
Supplement: Supplementary file 1 — (PDF 432 kb) [file 436_2021_7113_MOESM1_ESM.pdf]

XXX Reunión del Grupo Español de Dermatología Estética y Terapéutica de la AEDV (GEDET)
Donostia/San Sebastián, 15-17 de Noviembre de 2018

Nº 48

Eficacia pediculicida de dos productos frente a adultos, ninfas y liendres de *Pediculus humanus capitis*.

AUTORES: Marta Bajona Roig[1], Gabriela Bacchini[1], Gemma Puig Algora[2]
INSTITUCIONES: 1. Departamento Médico, Ferrer Internacional; 2. Departamento I+D, Ferrer Internacional.

ANTECEDENTES:

La mayoría de tratamientos para la pediculosis demuestran ser eficaces en la eliminación de las formas móviles (adultos y ninfas) pero su eficacia es limitada con las liendres.

OBJETIVOS:

Determinar la eficacia pediculicida de dos productos comercializados frente a estadíos móviles y liendres del piojo de la cabeza o *Pediculus humanus capitis* a distintos tiempos de tratamiento.

MÉTODOS:

Por metodología de inmersión (Gallardo *et al.* 2012), se sumergieron formas móviles y liendres durante 5 minutos en dos productos pediculicidas: uno a base de escualano y otro a base de miristato de isopropilo. Se realizó un estudio adicional a 2 minutos de tratamiento con el producto con escualano en formas móviles. La mortalidad post-tratamiento de las formas móviles fue registrada a los 5, 15, 30, 60, 180 minutos y 18 y 24 horas; la de las liendres a los 7 y 14 días. Se utilizaron tres réplicas de 10 especímenes por cada tiempo de inmersión, tratamiento y control (agua).

RESULTADOS:

Tras **5 minutos** de tratamiento, la eficacia de ambos productos frente a **estadios móviles** (mortalidad media ± desviaciones estándar) fue del **100% ± 0** en ambos productos. En **liendres**, la mortalidad fue del **86% ± 16** con el producto con **escualano** y del 13,3% ± 5,8 con el producto con miristato de isopropilo (Tabla 1).
Tras **2 minutos** de tratamiento el producto con **escualano** provocó el **100% ± 0** de mortalidad en formas móviles. El tiempo de inmersión necesario para la paralización general de los piojos en el producto con escualano fue de media 1,27 segundos.
En relación al modo de acción del producto con escualano, se ha observado una ruptura del tracto digestivo por estrés osmótico ligado a la retención o pérdida de agua respectivamente (Figura 1).

Tabla 1. Eficacia frente a liendres: porcentajes de mortalidad junto con el porcentaje medio y la desviación estándar, tras 5 minutos de inmersión en el producto con escualano (OTC Antipiojos® Fórmula Total) y miristato de isopropilo respectivamente.

	Porcentajes de mortalidad medios (%) en liendres tras 5 minutos de inmersión	
	Producto con escualano	Producto con miristato de isopropilo
Media ± DE	86 ± 16	13,3 ± 5,8

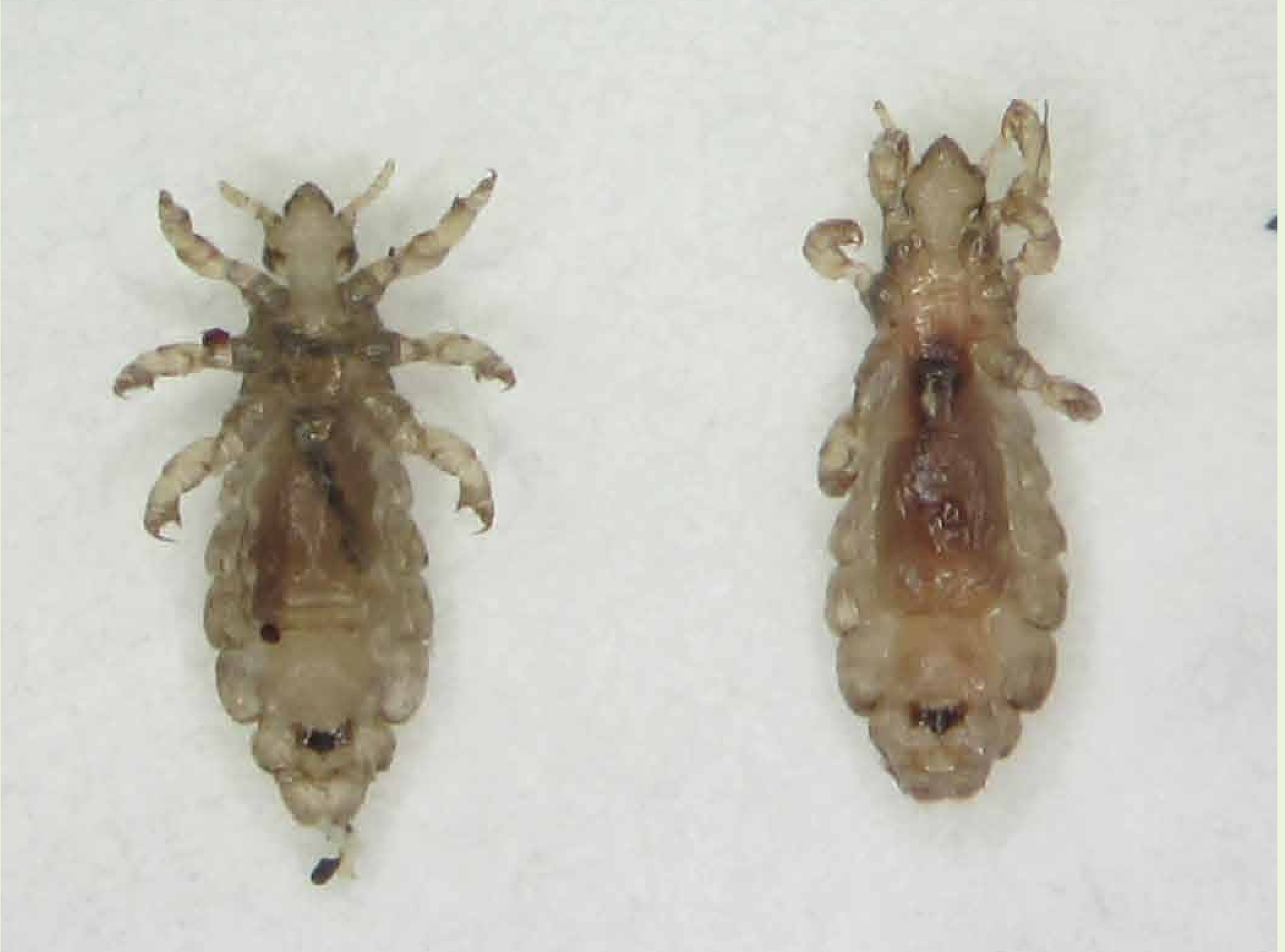

Figura 1. Espécimen adulto antes y después de 2 minutos de inmersión en producto con escualano (OTC Antipiojos® Fórmula Total).
Piojo sin tratar (control) (izqda.) y piojo tratado muerto (drcha.). Se ha observado una ruptura del tracto digestivo por estrés osmótico.

CONCLUSIONES

Es de vital importancia que un producto pediculicida posea excelente eficacia tanto en formas móviles como en liendres para un mejor control de la infestación. Aunque ambos pediculicidas demuestran una excelente eficacia (100%) en adultos y ninfas, el innovador producto a base de escualano supera en eficacia ovicida al producto con miristato de isopropilo (86% vs 13% respectivamente) en sólo 5 minutos de tratamiento.

Correspondencia:
Marta Bajona Roig
mbajona@ferrer.com

Gallardo, A., Mougabure-Cueto, G., Vassena, C., Picollo, M.I. & Toloza, A.C. Comparative efficacy of new commercial pediculicides against adults and eggs of Pediculus humanus capitis (head lice). Parasitology Research, 2012;110 (5):1601- 1606.

ferrer
