# Supplementary material for: Effectiveness and tolerability of a squalane and dimethicone-based treatment for head lice
Source: Parasitol Res. 2021 Apr 2;120(5):1883–90. doi: 10.1007/s00436-021-07113-y (PMC8084834; doi:10.1007/s00436-021-07113-y)
Supplement: Supplementary file 2 — (PDF 915 kb) [file 436_2021_7113_MOESM2_ESM.pdf]

infarma BARCELONA 2019
ENCUENTRO EUROPEO DE FARMACIA

CONGRESO EUROPEO DE OFICINA DE FARMACIA / SALÓN DE MEDICAMENTOS Y PARAFARMACIA
31ª Edición / Fira Barcelona - Gran Via / 19 mar - 21 mar 2019

A66

Mecanismo de acción de un nuevo pediculicida en formas móviles (adultos y ninfas tardías) y huevos (liendres) del piojo de la cabeza, *Pediculus humanus capitis*

AUTORES: BAJONA ROIG, M.[1]; BACCHINI, G.[1]; PUIG ALGORA, G.[2]

INSTITUCIONES: 1. Departamento Médico, Ferrer Internacional, Barcelona; 2. Departamento I+D, Ferrer Internacional, Esplugues de Llobregat, Barcelona

INTRODUCCIÓN:

El nuevo pediculicida a base de escualano provoca ruptura del tracto digestivo por estrés osmótico en piojo adulto[1]. Sin embargo, no se ha podido confirmar aún su mecanismo ovicida.

OBJETIVOS:

Establecer el mecanismo de acción de la fórmula pediculicida a base de escualano en estadíos móviles (adultos y ninfas tardías) y huevos (liendres).

MATERIAL Y MÉTODOS:

Metodología por inmersión basada en bibliografía científica[2,3]. La circulación del producto a través del cuerpo de ejemplares móviles y liendres se estudió utilizando colorantes disueltos en el producto en estudio (colorante Sudan Red 7B para ejemplares móviles; colorantes Sudan Red 7B y Sudan Blue II para liendres). Se realizó adicionalmente un control positivo con ejemplares móviles utilizando etanol absoluto con colorante Blue Brilliant. Se realizaron inmersiones durante distintos tiempos. Lavado posterior, examen bajo lupa binocular y fotografía.

RESULTADOS:

Tras 2 minutos de inmersión de formas móviles, el producto penetra en el sistema respiratorio a través de los espiráculos y difunde por el sistema traqueal **(Figura 1 A)**. La coloración parcial de la cabeza indica que el producto es capaz de fijarse a través de la epicutícula del exoesqueleto. La inmersión de liendres muestra como el producto recubre uniformemente la sección del opérculo correspondiente a los aerópilos y la sutura que une el opérculo con el cuerpo de la liendre **(Figura 1 B)**.

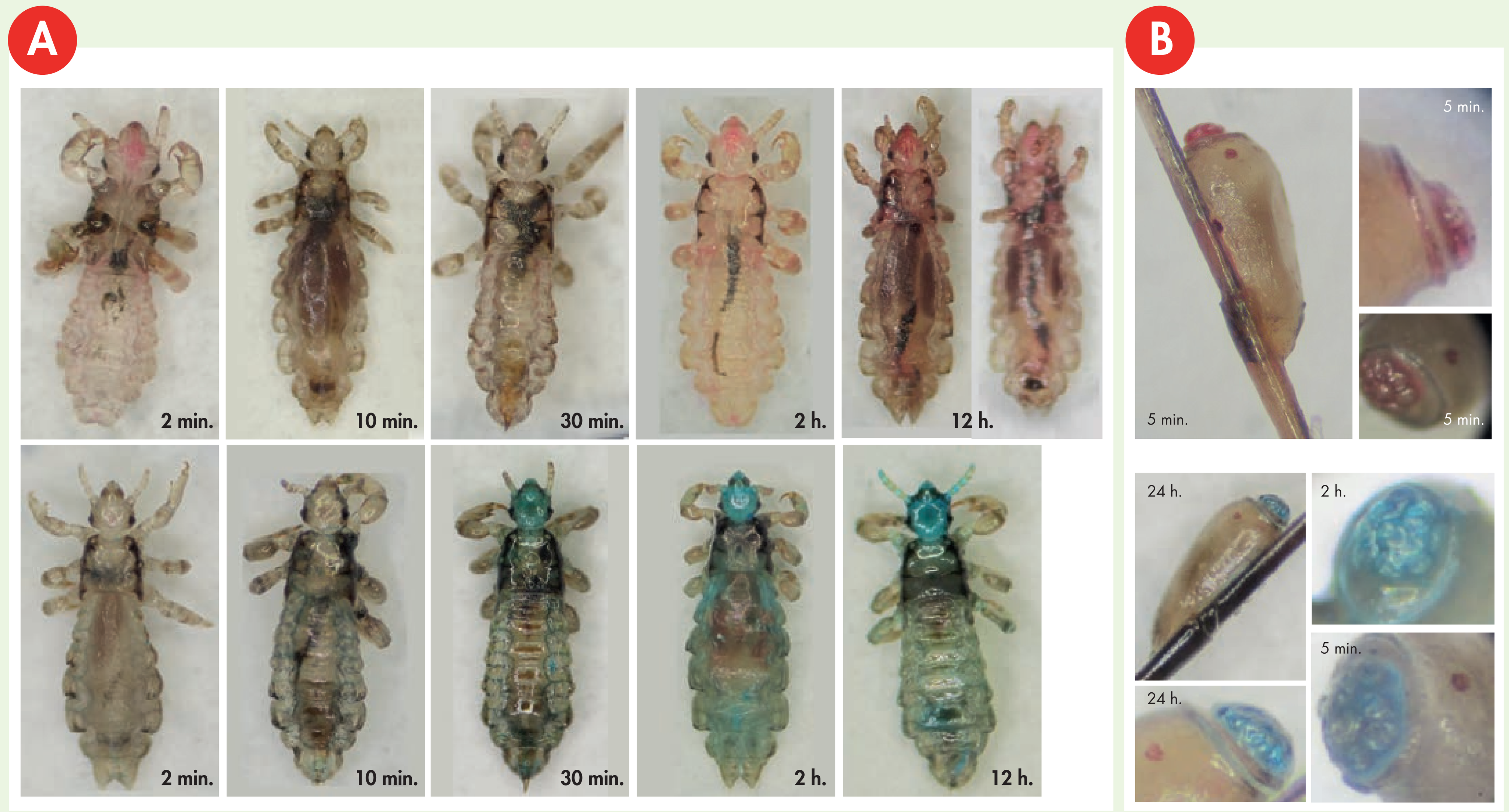

Figura 1. Apariencia del trazado de los productos a través del cuerpo del piojo **(A)** y liendres **(B)** a distintos tiempos de inmersión con distintos colorantes.

DISCUSIÓN:

Comprender el mecanismo de acción pediculicida y ovicida ayuda a desarrollar fórmulas cada vez más eficaces.

CONCLUSIONES

En estadios móviles, la fórmula pediculicida a base de escualano actúa bloqueando las estructuras respiratorias (espiráculos y tráquea) y penetrando a través de la cutícula del exoesqueleto. En liendres, el producto actúa penetrando por los aerópilos del opérculo hasta llegar al complejo de membranas internas que rodean al embrión impidiendo su desarrollo y provocando la muerte. En ambos casos el producto provoca la muerte de los insectos mediante desajustes metabólicos, deshidratación y asfixia.

Correspondencia: Marta Bajona Roig / mbajona@ferrer.com

Bibliografía:
1. Bajona Roig M, Bacchini G, Puig Algora G. Eficacia pediculicida de dos productos frente a adultos, ninfas y liendres de Pediculus humanus capitis. Póster presentado en XXX Reunión del Grupo Español de Dermatología Estética y Terapéutica de la AEDV (GEDET). Donostia/San Sebastián, 15-17 de Noviembre de 2018.
2. Gallardo A, Mougabure-Cueto G, Vassena C, Picollo MI, Toloza AC. Comparative efficacy of new commercial pediculicides against adults and eggs of Pediculus humanus capitis (head lice). Parasitol Res. 2012 May;110(5):1601-6.
3. Candy K, Brun S, Nicolas P, Durand R, Charrel RN, Izri A. Do drowning and anoxia kill head lice? Parasite.2018;25:8.
